# Editorial: The microbiome in cancer therapy response

**DOI:** 10.3389/frmbi.2026.1815455

**Published:** 2026-04-21

**Authors:** Elizabeth M. Park, Liza Makowski, Katherine L. Cook

**Affiliations:** 1 University of Texas Southwestern Medical School, Dallas, TX, United States; 2 Division of Hematology/Oncology, Department of Medicine, The University of Tennessee Health Science Center, Memphis, TN, United States; 3 Department of Cancer Biology, Wake Forest University School of Medicine, Winston-Salem, NC, United States

**Keywords:** cancer, immunotherapy, microbiome, microbiome & dysbiosis, obesity

Cancer therapy outcomes are increasingly understood to be the result of complex and dynamic interactions among tumor-intrinsic features, host immunity, metabolic state, and environmental exposures (Morad et al., 2021; Park et al., 2022; Arnone et al., 2024; Bohm et al., 2024; Lam et al., 2024; Bohm et al., 2025; Raji et al., 2025; Hebert et al., 2026). Among these factors, the microbiome has emerged as a key regulator of cancer risk, tumor progression, and therapeutic response, particularly in the context of immunotherapy (Park et al., 2022; Lam et al., 2024; Hebert et al., 2026). Rapid advances in sequencing technologies, experimental modeling, and translational research have strengthened associations between microbial composition and anti-tumor immunity, yet critical challenges remain in establishing causality, defining mechanisms, and translating insights into clinical practice.

This Research Topic brings together reviews and original research that collectively examine how microbiome-driven processes intersect with cancer immunity, metabolism, and therapeutic response. Rather than focusing on a single cancer type or intervention, these contributions highlight shared biological principles and experimental approaches that are shaping microbiome-informed oncology.

A central theme across this Topic is the interaction between the microbiome and anti-tumor immunity. One review by El-Derby et al. introduces immune-reactive tumor organoids as an innovative *ex vivo* platform to investigate how microbial metabolites influence cancer immunity and immunotherapeutic efficacy. By incorporating tumor cells, immune components, and microbiome-derived factors, this system addresses key limitations of conventional *in vitro* models and provides a tractable approach for patient-specific mechanistic studies. The application of such platforms represents an important step toward personalized assessment of microbiome–immune–tumor interactions and treatment prediction.

Complementing this mechanistic focus, Bailey et al. explore the influence of gut and lung microbiomes on lung cancer progression, immune responses, and therapeutic outcomes. Evidence supports a gut–lung axis in which microbial signals modulate systemic and local immunity, shaping the tumor microenvironment and influencing response to chemotherapy, targeted therapy, and immunotherapy. Alterations in microbial diversity and composition modulate immune cell populations, including T cells, dendritic cells, and macrophages, highlighting opportunities for microbiome-targeted interventions to enhance treatment efficacy.

Obesity and metabolic dysfunction are featured as additional recurring themes throughout this Topic. Original research extends the concept linking metabolic dysfunction and microbial dysbiosis by demonstrating that obesity profoundly alters both the normal pancreatic and pancreatic cancer microbiomes in mice and humans. Using 16S rRNA sequencing, da Cruz et al. show that obesity accelerates pancreatic ductal adenocarcinoma progression, reduces microbial diversity, and alters bacterial community composition within pancreatic tissue, with obese mice exhibiting accelerated cancer progression and increased mortality compared to lean controls. Microbial changes are accompanied by increased circulating pro-inflammatory cytokines and altered tumor immune infiltrates, suggesting that metabolic dysregulation may modulate tumor immune environments through microbiome-dependent mechanisms.

At the interface of microbiome modulation and metabolism, original research by Yang et al. on the microbial metabolite mycosubtilin demonstrates its capacity to reduce weight gain, modulate lipid metabolism, and alter gut microbiota composition in mice. Although not directly cancer-focused, these findings are highly relevant given the links between obesity, immune dysregulation, and cancer risk, highlighting the potential of targeted microbial metabolites as modulators of host metabolism and immunity.

Finally, a review by Kamassa et al. examining oral and vaginal microbiomes in HPV-related cervical, head, and neck cancers emphasizes mechanisms by which microbial dysbiosis may modulate viral persistence, immune evasion, and carcinogenesis. By highlighting the critical need for microbiota profiling and mechanistic research in sub-Saharan Africa, where unique socio-economic and environmental factors shape microbial communities and cancer burden, this work expands the scope of this Research Topic to global health contexts and underscores the importance of geographically inclusive microbiome research.

Collectively, the articles in this Research Topic position the microbiome–immunity–metabolism axis as a central regulator of cancer biology including both cancer risk and progression to therapeutic response. These contributions emphasize the context-dependent nature of microbiome effects and the necessity of integrative, mechanistically informed approaches. Continued progress will rely on leveraging advanced experimental models, standardized methodologies, and inclusive research strategies to translate microbiome insights into effective, equitable cancer therapies.

## References

[B1] Arnone A. A. Wilson A. S. Soto-Pantoja D. R. Cook K. L. (2024). Diet modulates the gut microbiome, metabolism, and mammary gland inflammation to influence breast cancer risk. Cancer Prev. Res. (Phila. Pa.) 17, 415–428. doi: 10.1158/1940-6207.CAPR-24-0055, PMID: 38701438 PMC11372361

[B2] Bohm M.S. Joseph S. C. Sipe L. M. Kim M. Leathem C. T. Mims T. S. . (2025). The gut microbiome enhances breast cancer immunotherapy following bariatric surgery. JCI Insight 10, e187683. doi: 10.1172/jci.insight.187683, PMID: 40272913 PMC12220976

[B3] Bohm M. S. Ramesh A. V. Pierre J. F. Cook K. L. Murphy E. A. Makowski L. . (2024). Fecal microbial transplants as investigative tools in cancer. Am. J. Physiol. Gastrointest. Liver Physiol. 327, G711–G726. doi: 10.1152/ajpgi.00171.2024, PMID: 39301964 PMC11559651

[B4] Herbert J. R. Murphy E. A. Playdon M. C. Makowski L. Ibele A. R. Fairman C. M. . (2026). Are GLP-1 receptor agonists a ‘magic bullet’ for cancer? Nat. Rev. Cancer 26, 3–4. doi: 10.1038/s41568-025-00874-z, PMID: 40931125 PMC13291921

[B5] Lam T. K. Daschner P. Ishibe N. Wali A. Hall K. Czajkowski S. . (2024). Metabolic Dysregulation and Cancer Risk Program (MeDOC): a transdisciplinary approach to obesity-associated cancers. J. Natl. Cancer Inst. 116, 1555–1561. doi: 10.1093/jnci/djae134, PMID: 38885413 PMC11461156

[B6] Morad G. Helmink B. A. Sharma P. Wargo J. A. (2021). Hallmarks of response, resistance, and toxicity to immune checkpoint blockade. Cell 184, 5309–5337. doi: 10.1016/j.cell.2021.09.020, PMID: 34624224 PMC8767569

[B7] Park E. M. Chelvanambi M. Bhutiani N. Kroemer G. Zitvogel L. Wargo J. A. . (2022). Targeting the gut and tumor microbiota in cancer. Nat. Med. 28, 690–703. doi: 10.1038/s41591-022-01779-2, PMID: 35440726

[B8] Raji L. M. Siddique M. M. Bohm M. S. Pierre J. F. Playdon M. C. Summers S. A. . (2025). Murine models of obesity-related cancer risk. Cancer Prev. Res. (Phila. Pa.) 18, 509–529. doi: 10.1158/1940-6207.CAPR-24-0545, PMID: 40509936 PMC12289351

